# Exploring the barriers and facilitators to volunteering as an intervention for those with long‐term neurological conditions: How make therapeutic volunteering possible?

**DOI:** 10.1111/hex.13891

**Published:** 2023-10-15

**Authors:** Helene Eisenhut, Johnny Collett, Farzaneh Yazdani

**Affiliations:** ^1^ Faculty of Health and Life Sciences Oxford Brookes University Oxford UK; ^2^ Centre for Movement, Occupation and Rehabilitation Sciences Oxford Brookes University Oxford UK

**Keywords:** chronic disease, focus group, intervention development, social inclusion, volunteering

## Abstract

**Background:**

Volunteering may have therapeutic benefits, but little is known about its requirements and potential for people with neurological conditions (pwNC).

**Design:**

Two separate focus groups were conducted in Darmstadt, Germany: one group consisting of six pwNC and another group consisting of four health care professionals and three volunteering service providers. The focus groups were audio‐recorded, transcribed and data were managed using NVivo12. The thematic analysis was applied.

**Results:**

Four main themes were identified: (1) Impact of volunteering, (2) Individualisation, (3) Developmental space and (4) Funded supported volunteering.

**Conclusion:**

According to the findings, volunteering can be used as a strategy to enhance physical, mental and social well‐being in disease management for people with long‐term neurological conditions.

**Patients or Public Contribution:**

Facilitators for accessibility of therapeutic volunteering; involvement of pwNC.

## 1 INTRODUCTION

Volunteering, defined as free and unpaid activity (outside of the family) through an organisation,^1^ may have potential benefits through facilitating social and meaningful activities for people with neurological conditions (pwNC).^2^, ^3^, ^4^, ^5^, ^6^ According to the International Classification of Functioning, Disability and Health (ICF), participation is an outcome of a variety of factors that need to be considered in enhancing the health and well‐being of people with impairment. Participation can be restricted by physical, social and attitudinal environmental factors. The extent of interference in the performance of activities is therefore not determined merely by the health problem but by the factors of facilitators or barriers in the environment.^7^

Studies have identified the potential of volunteering to facilitate social participation and improve self‐confidence, self‐efficacy and well‐being through reduced social isolation and by providing a new social role.^1^, ^3^, ^6^ One significant factor in developing self‐efficacy is others' feedback on one's performance. An appropriate level of self‐efficacy leads to higher motivation and engagement to participate in activities that would lead to changes in one's life.^8^ Volunteering has the potential to enhance pwNCs' self‐efficacy and also potentially the level of engagement with participation in activities and social participation. However, attitudes towards pwNCs have been identified as a barrier to volunteering, and pwNCs are perceived as receivers of volunteering services rather than contributors.^9^ Shandra^10^ found that while pwNC can and do volunteer, neurological conditions were negatively associated with formal volunteering, and a better understanding of inequalities and identification of opportunities is required.

Designing a volunteering intervention is complex due to the number of components and considerations involved; stakeholder engagement is crucial to its development.^11^ The perspective and experience of patients and those who might be involved in delivering a volunteering intervention are lacking in the literature.^12^ The perspectives of pwNC on how to overcome barriers associated with participating with volunteering in the community as well as the perspectives of health care professionals and those who provide volunteering opportunities are not known. In Germany, approximately 1.7 million people receive disability pensions due to orthopaedic, neurologic, psychologic/psychiatric or tumour diseases. However, access to volunteering and research to inform volunteering programmes is limited.^13^

The study sought to gain insights into the experiences and perspectives of pwNC and encourage volunteering service providers and health care professionals in Darmstadt, Germany, to identify potential facilitators and inform on organisational structures and resources required to deliver an intervention.

## 2 METHODS

### 2.1 Study design

This qualitative study used focus groups for data collection and was reported considering Consolidated Criteria for Qualitative Research guidelines.^12^ Focus groups were selected to create a discussion to exchange points of view. The moderator ensured that everyone's voice was considered, even in the case of a consensus. No idea was excluded.^13^ Four relevant stakeholders were identified: pwNCs, volunteering service provider, outpatient care and occupational therapist (OT). To regulate the dynamic of interaction, two focus groups, one comprised of pwNC and the other comprised of professionals, were conducted between January and April 2020, and both met twice.^14^ Thematic analysis was used. Ethical approval was obtained (Oxford Brookes University: 191339), and all participants gave written informed consent.

### 2.2 Recruitment

PwNC were recruited via an advert distributed by information centres and self‐help groups in Darmstadt, Hessen, Germany. Health care professionals and volunteering service providers were recruited in the same city through publicly available contact details on organisational websites.

### 2.3 Participants

Eligibility criteria are presented in Table 1; neither the health professionals nor the volunteering service providers were expected to have experience supporting clients in accessing volunteering services.

**Table 1:** Inclusion/exclusion criteria for participants.

Inclusion criteria for pwNC	Exclusion criteria for pwNC
1. Adults between 18 and 64 years of age with a diagnosis of a chronic neurological condition.	1. Ex‐clients of the researcher's practice.
2. Level of disability of 50 with a full reduction in earning capacity or a care level of 1 or 2 according to section 152 of Book IX and section 15 of Book XI of the German Social Security Code (SGB IX + XI).	2. Difficulties communicating and holding a conversation.
3. Essential problems (present less than 1–3 times a week) according to the International Classification of Functioning, Disability and Health in the area of activities and participation in the domains of learning and applying knowledge (d1), general tasks and demands (d2), communication (d3), mobility (d4), self‐care (d5), domestic life (d6), interpersonal interactions and relationships (d7), community and social and civic life (d9).	
Inclusion criteria for health professionals	
1. Registration on a website.	
2. Two or more years of experience with clients with a full reduction in earning capacity and/or care level 2.	
Inclusion criteria for volunteering service provider	
1. One or more years of experience in the placement and supervision of volunteers.	

Abbreviation: pwNC, people with neurological condition.

### 2.4 Focus groups

Considering the small size of the population, a purposive sampling method was used. The total sample size was not predetermined.^15^ The number of meetings for each focus group was decided based on data saturation, which was reached after two focus group meetings for both professional and patient groups.^16^

Focus groups were held at an occupational therapy outpatient practice in Darmstadt, Germany, and scheduled outside opening hours. Each lasted approximately 90 min (with breaks as required). The first author conducted the focus group interviews with a co‐moderator. The moderator guided the discussion and took notes and the co‐moderator took over at specific points. The moderator has 22 years of experience as an OT and the co‐moderator has approximately 16 years of work experience managing and delivering workshops. Neither the moderator nor the co‐moderator knew any participants before the focus groups. The moderator was previously associated with one of the OTs and one volunteering service provider.

The focus group moderators sought to create a suitable environment to enable people to freely share their perspectives at an individual's pace of speech. The first meetings of each focus group commenced with ‘ice‐breaking’ activities like drinking tea, eating finger food and small talk about how the participants arrived to establish confidence and create a trusting environment. After that, a semistructured discussion was initiated, using the funnel design, moving from broad to narrow topics.^17^ See Table 2 for the topic list of the focus groups.

**Table 2:** Topic list of the focus groups.

Interview topics	Questions: Group of pwNC	Questions: Group of professionals
Initial brainstorming	Do you know or can you imagine factors that hinder people with neurological chronic illness, unemployable, from volunteering?	Do you know or can you imagine factors that hinder people with neurological chronic illness, unemployable, from volunteering?
Barriers and facilitators of volunteering	Can you imagine factors like specific personal characteristics or circumstances of the physical or social environment that helped you to volunteer or hindered you from volunteering?	Can you name environmental or personal characteristics or occupational requirements that could make access for neurological chronically ill people easier?
Content of a volunteering intervention	What are your needs regarding the development of the match between volunteer posts and neurological chronically ill unemployable candidates?	How may services be improved to support people with neurological chronic illness to have access to volunteering?
Way of delivering	If volunteering becomes an intervention, what support would you find helpful to access volunteering?	If volunteering becomes an intervention, what support would you find helpful to access volunteering services?
The benefit of intervention	What benefits or burdens of volunteering do you expect?	What benefits of volunteering do you expect? What burden of volunteering do you expect?

Abbreviation: pwNC, people with neurological condition.

The second meeting of each group included follow‐up questions related to the preceding questions, a discussion of the facilitators and barriers discussed by the other group to obtain confirmation or dissenting opinions and suggestions for problem‐solving. In the pwNC focus group, the mini‐team‐debate technique was also used. The moderators pursued the strategy of bringing all viewpoints in focus and discuss further; consensus was not required. Any idea that needed further exploration was brought up in the second focus group to support further clarification. After 2 focus groups, the information seemed to be coherent, and no further clarification was needed. Thus, saturation was deemed to have been reached, and there was no need for further meetings.^16^

### 2.5 Data analysis and synthesis

The author transcribed the digitally audio‐recorded data verbatim for analysis.^18^ To identify and develop themes in the data, thematic analysis was conducted following the concepts of Braun & Clarke^19^ and using the ‘interpretative‐reductive analysis’ of Lamnek.^20^ Both inductive and deductive approaches were used. According to the conducted focus groups, the initial questions and data were assigned to themes derived from the scoping review to establish the focus group function.^17^ The discussion revealed the expansion and depth of the topic; hence, further themes could be identified that did not exactly fit into the background knowledge of the theory. Data were managed using NVivo12 software.

New themes were developed when data showed no assignability, determining their main statement.^19^ Every sentence or section of data was reviewed, whether they were assignable to existing themes or if a new one had to be created. In the case of two similar themes, a merger with a generic term was considered. The final themes are based on the second meetings of both focus groups, not the first one. See a comparison of the themes derived from the scoping review and focus groups in Table 3 and the frequency of references assigned to each theme derived from the focus groups in Table 4. The frequency of references for each theme was compared between professional and pwNC focus groups using *χ* ^2^ and post hoc *z* statistics, *α* .05.

**Table 3:** Themes derived from the scoping review and the focus groups.

Themes derived from the scoping review	Barriers to volunteering for pwNC, definitions of volunteering, the interplay of health and social inclusion, the character of pwNC who volunteer, opportunities of volunteering, coping strategies of pwNC, implementation strategies of new interventions.
Themes derived from the focus groups	Facilitators of volunteering, profiles of pwNC who volunteer, volunteering as an intervention, individual biographies of access to volunteering, (critical) perspectives on inclusion, impact, volunteering requirements.

Abbreviation: pwNC, people with neurological condition.

**Table 4:** Frequency of references assigned to each theme.

Themes	Number of coded references	pwNC	Professionals	Diff
Profiles of chronically ill people who volunteer	56	32	24	*p* = .135
Overcoming barriers to volunteering	47	20	27	*p* = .332
Volunteering as an intervention	40	12	28	*p* = .008
Individual biographies of access to volunteering	16	12	4	*p* = .028
(Critical) perspectives on inclusion	14	7	7	*p* = .920
Impact	13	10	3	*p* = .035
Requirements of volunteering	11	3	7	*p* = .213

Data were considered according to the perspectives and experiences of those with chronic neurological conditions, health professionals or volunteer service providers. The ‘thematic networks’ of attride‐stirling were used to aid the analysis and demonstration of the findings.^21^ The analysis process was conducted from December 2020 until April 2021 and was nonlinear and recursive, with frequent reviews while including personal memoranda alongside field notes.

Focus groups were conducted in German, and illustrative quotes are reported in the main text translated into English (for German, see Supporting Information 1). To ensure correct translation, quotes were discussed with an independent bilingual person and a research team for technical translation.

## 3 RESULTS

### 3.1 Participants

Thirteen people were recruited, *n* = 6 pwNC and *n* = 7 professionals (*n* = 4 health professionals (*n* = 2 outpatient nurses, *n* = 2 OTs) and *n* = 3 volunteering service providers).

From the pwNC group, one participant did not attend the second meeting because of illness. In the professional group, one participant (volunteer service provider) did not attend the first meeting due to travel problems. Two outpatient nurses could not attend the second meeting due to work and personal commitments. The COVID‐19 pandemic in Germany coincided with the professionals' second focus group. As a result, telephone and video conferencing were utilised by *n* = 2 professionals.

PwNC had the following diagnoses: multiple sclerosis, brain haemorrhage, traumatic brain injury, polyneuropathy and restless legs syndrome. There was no difference between professional and pwNC focus groups in terms of gender *p* = .415 or age *p* = .268. However, a greater proportion of professionals were university‐educated *p* = .033. Biographical data are shown in Table 5.

**Table 5:** Biographical data of the participants.

Characteristics	Group of chronic ill volunteers *n* (%)	Group of volunteering jobs provider *n* (%)	OT and ON	Nonvolunteer
Age	48, 51, 55, 60, 71	26, 37, 77	OT: 43, 43 ON: 55, 60	60
Gender	4 Women, 1 man	2 Women, 1 man	4 Women	1 Man
Level of disability	GdB 50, 60, 70, 90, 100			GdB 50
Years of voluntary work	7, 7, 10, 21, 40	7, 5, 5	Years of professional experience OT: 17, 14 ON: 35, 40	
Hours of volunteering per month	2–3, 10–12, 15, 5–40, 40	5–25, 8–10, 0–40		
Social support	Yes: 4 No: 1			No: 1
Living alone	Yes: 1 No: 4	Yes: 1 No: 2		Yes: 1
Education level	High school graduation: 3 Vocational qualification: 3 University degree: 2	University degree (3)		University degree: 1
Employed		Yes: 2 Pensioner: 1	Yes: 4	
Car owner	Yes: 3 No: 2			No: 1
House owner	Yes: 1 No: 4	Yes: 1 No: 2		No: 1

Abbreviations: ON, outpatient nurses; OT, occupational therapists.

### 3.2 Predominant themes

**Figure 1:**
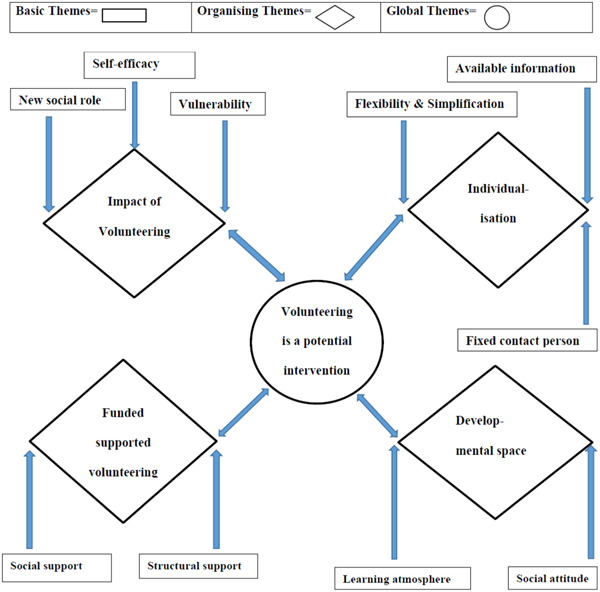
Thematic network.

The initial themes identified were as follows: Facilitators of volunteering, profiles of pwNC who volunteer, volunteering as an intervention, individual biographies of access to volunteering, (critical) perspectives on inclusion, impact and volunteering requirements. The frequency of reference assigned to each theme was significantly different between pwNC and professionals (*p* < .01). The post hoc *z*‐test found that professionals more frequently referenced ‘volunteering as an intervention’ (*p* = .008) and pwNC more frequently referenced ‘individual biographies of access to volunteering’ (*p* = .028) and ‘impact of volunteering’ (*p* = .035). The frequency of references assigned to each theme is shown in Table 4. The thematic networks are shown in Figure 1.

### 3.3 Thematic network: Basic, organising and global themes

The thematic network presents the found themes interconnected, without hierarchy, in a fluid way. The basic themes include themes that pop up in the background. Collections of basic themes were brought into context by their assignment to an organising theme. The organising themes highlight the focal points of the focus groups. The concluding theme in the data is showed as the global theme.^21^

### 3.4 The basic themes

Basic Theme 1: Self‐efficacy

The main impact of volunteering for pwNC is to do something you are good at and show it to others.

Volunteering means you can do what you can do. (Nr. C2)

Five of the six participants were already volunteering. One became ill as an adolescent, and she started volunteering soon. One in the group was self‐employed, an entrepreneur when he became sick. He had to give up bicycling, his favourite leisure activity. Subsequently, he found specially equipped bicycles and started organising tours for big groups.

I lost cycling with the onset of my illness. Cycling had always been crucial for me […], I went to the fair in [town] and discovered the special bikes, the recumbent bikes […] I was so enthusiastic about it and then founded the private initiative [name]. (Nr. C1)

The other three participants started volunteering for a self‐help group related to their diagnosis to support the group operation. See Table 6 for the volunteering area that the pwNC currently participates in. One of the pwNC described her volunteer job as similar to her professional activity in the past.

**Table 6:** Voluntary work of chronically ill participants.

Volunteering activities	Helping in a hospital, helping in a deaconry office, distributing food to homeless people, organising bike tours for people with/without disability, supporting a self‐help group with submitting applications, engaging in public relations, board activity or organising regulars' tables, providing cognitive training, providing language courses in German for people seeking asylum.

One of the volunteering providers emphasised the sense of achievement that one feels at the end of a lot of work.

Of course, sometimes it's fun when you see the results […] A lot has to be done in advance before you really meet together. I find that amazing. (Nr. P5)

Basic Theme 2: New social role

The pwNC group highlighted the inclusive aspect of volunteering, allowing transition from getting assistance to assisting.

I think we would be doing a great service to society if someone came to do voluntary work and made a contribution […] it would perhaps change the social image of people with disabilities a little bit. (Nr. C3)

Basic Theme 3: Vulnerability

The outpatient nurses warned about the misuse of volunteering in the health system, especially in nursing care.

Yes, I think that the state rests too much on the fact that you could use volunteers for certain tasks because it's just cheaper. (Nr. P5)

In line with society's pace of life, one pwNC encouraged society to adapt and learn.

My speed at the moment I do something [sic] should be allowed to be my speed, and that would be good for society as a whole if we all slowed down a bit. (Nr. C1)

Basic Theme 4: Flexibility and simplification

To meet their needs, pwNC discussed how volunteering could be more flexible. The starting point was adaptable time commitment. The OTs agreed:

They might say I can do half an hour a day or an hour a week, or I want three hours a day because I need the structure. […] That these people [pwNC] don't feel this pressure from the beginning, like in the real working world. (Nr. P2)

One of the volunteering providers described how the tasks of volunteering could be divided in terms of severity and location so that people with physical limitations can be a part of the volunteer group from home.

Especially in spring, there is the possibility to grow plants on the windowsill, to do it for yourself, but to know what you are doing it for [sic]. (Nr. P5)

Basic Theme 5: Available information

PwNC noted that there is often a lack of information about volunteering that is crucial for their decision‐making process. They wondered if the volunteer service provider could better signal its flexibility and provide more information about time aspects, accessibility and required skills.

They can also make descriptions of their work under other aspects so far, but there you might have to add fields like ‘flexible in terms of time’ or ‘up to three hours per week’, ‘barrier‐free’, or ‘without a lift’ or ‘you have to be able to do this and that!’ (Nr. C3)

Basic Theme 6: Fixed contact person

To have a fixed contact person was a suggestion made by three different parties. The group of pwNC found that an application procedure could be simplified by having one contact person. Anticipating accompanying pwNC to find the right volunteering job, the OTs wished to have a contact person to communicate the expectations of the volunteering service provider and to receive feedback about the performance of pwNC over time. The volunteer service provider said that they would need someone, such as an OT, to bridge the gap between themselves and the pwNC.

I think it always comes down to having a coordinator you can talk to. (Nr. C3)

Basic Theme 7: Social attitude

Discussing whether access to volunteering for pwNC is about justice or social attitude, the volunteer service providers pointed out that volunteers are generally not necessarily socially minded in the sense of helping pwNC. One of the volunteer service providers anticipated the greater effort to support pwNC and requiring effort beyond what could be managed by another volunteer. Appraisals, guidance, support or supervision of pwNC could be offered occasionally, not regularly. On the one hand, they agreed that everyone has the right to volunteer, but on the other hand, they found that additional resources are necessary.

Putting yourself in their situation was suggested as a way to make volunteering accessible by pwNC.

For me as a disabled person, it is more important […], that you think about the fact that you can get into this situation yourself. (Nr. C4)

Another person felt that it was important for people to be more appreciative of each other, and one should not deny pwNC their potential.

I think appreciation is critical […] we should appreciate each other […] I have a past, I have a lot of know‐how, I have a lot of potential from my work so far, which is hidden, which I may not be able to access at the moment. (Nr. C1)

Basic Theme 8: Learning atmosphere

PwNC discussed learning in the context of expectations and challenges.

Don't challenge the beginning [sic] too much by setting the requirements so high, but start with smaller requirements, but then also ‘throw the child in at the deep end and say, now do this, and then we'll see if we can build on it’. (Nr. 4)

Basic Theme 9: Social support

PwNC believed that appreciation, respect, confidence, enough time for private life and no pressure of envy from fellow sufferers supported volunteering. Conversely, limitations to the physical and psychological condition were perceived as barriers for professionals. PwNC discussed having a team who can stand in at short notice when individuals experience adverse psychological fluctuations.

The solution always requires a group or a supporter. (Nr. C6)

Basic Theme 10: Structural support

PwNC discussed a low‐stimulus environment, expense allowance and transport service for people with mobility difficulties and suggested mechanisms to reduce pressure.

This pressure of having to deliver a certain performance, of being measured by it, then the pressure is gone. (Nr. C1)

### 3.5 Organising themes

The findings of this study are presented in four organising themes.

Organising Theme 1: Impact of volunteering

Intrinsic factors that promote or inhibit volunteering (basic themes: self‐efficacy, new social role, vulnerability).

The pwNC argued that many challenges are common to volunteering in general.

Then we come to the disadvantages […] envy and jealousy have already been mentioned, but then also the demand for expertise and energy, partly also financial expenditure, too […] that one is left alone with the tasks so that it comes to overload […], but I'm afraid that it's a problem of voluntary work in general and that it doesn't just apply to the chronically ill. (Nr. C1)

Some of the pwNC and volunteering service providers talked about bearing a considerable weight of responsibility. PwNC reported stress associated with cancelling with short notice, feeling overloaded and lack of plannability and the inherent dependence on volunteers. They felt that volunteers have to be willing to commit substantial time and potential personal costs. However, some pwNC stated that volunteering provides an opportunity to demonstrate their abilities without the consequences of failing in paid work.

PwNC agreed about the unpredictability of performance and being able to commit occasionally. However, they needed to be perceived in terms of their intention to give their 100% and defend themselves against the assertion of unreliability, which is not unique to pwNC.

But there are also unreliabilities [sic] in healthy people. I know some people […] who have more absences than working hours. (Nr. C5)

The professionals wondered if pwNC would agree to make their limits transparent so that volunteering services know what to expect.

Organising Theme 2: Individualisation

Conditions to match volunteer opportunities to abilities (basic themes: flexibility and simplification, available information and fixed contact person)

The professionals identified the conditions and characteristics of volunteering jobs to give access to pwNC. They found a simplified application procedure; the opportunity to confirm or to cancel at short notice, as well as having the choice of group work, working alone or just being present, could be helpful. Important information, identified by pwNC and professionals, concerned the variety of volunteering jobs, dates, meeting places, accessibility, the required time and required skills. An online platform was believed to be the best way to provide this information and trial periods, recommended in the case of a mismatch.

The professionals suggested volunteering jobs near the residence of the pwNC; time flexibility and choice of tasks could be helpful. Using a buddy system or a permanent contact person could also be supportive. However, the OTs' responsibility of ensuring against excessive demands and that pwNC did not feel left alone was also noted.

And that it can reach a level relatively quickly, depending on what you want to offer, where you say: ‘Oops, that has nothing to do with “part‐time” anymore. This is a real job that you do on the side. Not everyone can afford that’. (Nr. P5)

Organising Theme 3: Developmental space

The basic themes of social attitude and learning atmosphere influenced the developmental space of considering volunteering as a therapeutic intervention.

PwNC pointed to the importance of considering the difficulty of the task.

You always have to find out that you don't set the tasks too high or too low. So that they [the tasks] can still offer satisfaction and success. (Nr. C5)

Volunteers should not feel exploited, and activities must be relevant but not targeted solely at therapeutic skills training. They recommended no minimum participation requirement, cooperation on an equal footing, an inclusive attitude and encouragement towards new group members and a contact person in each group. Some of the group found it essential to have a low turnover of members.

Organising Theme 4: Funded supported volunteering

This theme consists of the basic themes of social and structural support. Volunteering service providers discussed the need for political, legislative and funding support. This could establish a project office to support pwNC, such as helping them try different volunteering jobs.

But it would also be nice if there was someone from the community […] because the service provider himself can't always afford to guide the people. (Nr. P2)

The professionals commented that OTs could help network with family members or friends of the clients to support their participation in volunteering, reducing the workload.

This could provide an overview, create a pool of information and bring together volunteering organisations, therapists and pwNC.

The OTs concluded that supporting therapeutic volunteering would take longer than a 30–60‐min appointment. Implementation would need the agreement of management and other team members and would require greater flexibility in scheduling. However, implementation was feasible within current systems (in the locality) and is consistent with a doctor's prescription for occupational therapy. They noted that the therapeutic intervention costs may be greater than standard interventions.

### 3.6 Global theme

The global theme summarised data regarding volunteering as a potential intervention.

The OTs appreciated the idea of therapeutic volunteering.

People might find it better to do something than to be placed somewhere where they play board games or things like that. That has been food for thought for me. (Nr. P2)

The volunteering service provider initially expressed reservations.

Is a person concerned at all willing to allow transparency, or does he prefer to keep his difficulties behind him. I see the potential for difficulties there. (Nr. P1)

The OTs discussed their potential role: ‘opening doors’, introducing volunteering opportunities, pairing the client's ability to the volunteering requirements and organising trials. The rest of the professionals agreed to OTs' suitability in this role.

The volunteering service also needs to consider the needs and expectations of those offering the job.

I think that it would help everyone. If you say: ‘Flexibility is not possible for me; I need reliable people’, then I know as someone who refers or recommends the job: ‘Okay, it's not suitable for this [person]’. (Nr. P3)

The OTs were optimistic that clients could gain independence in the process and that further support could be provided through ergonomic and environmental adaptations.

I think that taking care of the chronically ill person would probably be reduced to an adjustment of the workplace; rather, it would not even be necessary in case of doubt if we were to choose the right one [workplace]. (Nr. P3)

PwNC also felt that they could gain independence, only requiring initial training and perhaps providing some aid.

The OTs discussed and suggested approaches (considering therapeutic benefit) to match volunteering jobs with clients, for example, work shadowing (i.e., for being involved, getting a feeling of satisfaction) and undertaking a small task (i.e., for assessing one's condition). The volunteering service providers agreed and suggested precise definitions of roles, competencies and responsibilities between OTs and volunteer services. They believed that they might not have as much time as OTs and, considering the potential for diverse and complex problems, suggested that OTs could advise volunteering service providers. One volunteering service provider noted that organisations are small groups and inexperienced in assisting pwNC.

## 4 DISCUSSION

This study revealed intrinsic and extrinsic motivators and potential benefits of volunteering for pwNC. The findings confirmed participation restrictions, consistent with the ICF framework,^7^ and provided information to support the development of solutions to support pwNC in volunteering. The study revealed several themes; while organising Theme 1, ‘Impact of volunteering’, found challenges common to volunteering in general, organising Theme 3, ‘Developmentals space’, appeals to an inclusive attitude. Considering fluctuating physical performance, pwNC ask to acknowledge that they intend to give the maximum. Organising Theme 2 concerned the importance of the ‘Individualisation’ required to match volunteers' opportunities to the abilities of pwNC. Confirming the hesitation of volunteering service providers to include pwNC, organising Theme 4 concerned ‘Funded supported volunteering’, anticipating increased financial costs associated with supporting volunteering in pwNC.

PwNC emphasised the benefits of being valued and respected and improving self‐efficacy, consistent with social activities' benefits.^4^ According to the theory of Bandura, four factors form the basis for the belief in self‐efficacy: coping experiences, social modelling (regarding social learning), encouragement from others and physiological conditions.^8^ These four factors are facilitated by volunteering. The basic Theme 1, ‘Self‐efficacy’, and Theme 2, ‘New social role’, describe coping experiences when volunteering. Three themes imply encouragement from others and stimulating social modelling and learning by observing other volunteers acting: basic Theme 6 discusses the importance for pwNC of a ‘Fixed contact person’ and the involvement of an OT for a feedback loop, the basic Theme 8 highlights the need for a ‘Learning atmosphere’ and basic Theme 9 ‘Social support’ highlights the advantage of working in a team. Basic Theme 10 concerned ‘Structural support’ to reduce the fear of failure felt by pwNC, enabling an optimistic attitude.

Moreover, pwNC expressed the chance to change their role from being recipients of assistance to assisting others. In addition to these benefits, Okun et al.^22^ found that frequent volunteering was associated with longevity compared with people who did no or little volunteering.

Interestingly, none of the health care providers had experience volunteering for pwNC. Together, these results indicate that a greater awareness of potential benefits may be required.

The health consequences of social isolation are well researched.^23^ Occupational therapy aims to maintain and regain independence in everyday life and support people in engaging in activities that are meaningful to them.^24^ When there is an activity imbalance or deprivation, volunteering as an intervention could effectively fill the client's day with activity and provide meaning where the patient does something relevant for others in society.^25^ Certainly, the pwNC in the present study valued the volunteering that they took part in. Until now, OTs have used an interest checklist to activate past or new hobbies if the return to work is excluded. However, hobbies do not necessarily imply regular interaction with other people. Enabling pwNC to engage in voluntary work benefits the social environment and may be a helpful treatment option for OTs. Vaportzis et al.^26^ reported that recently, real‐world interventions have received increased interest. The provided activities can occur in community‐based or close‐to‐real settings, combining physical, cognitive and social activities. Volunteering could be a suitable setting for this kind of intervention.

Despite initial scepticism, volunteer service providers saw the value and expressed interest in the potential of volunteering for pwNC. Importantly, this study revealed several aspects from the viewpoint of volunteer service providers that should be considered. An individualised approach to matching the volunteering opportunity to the pwNC was seen as important, including having detailed information on the requirements. To facilitate this, a fixed contact person who could anticipate support needs was seen as advantageous. Indeed, the attitude of the social environment has been found to determine participation in social activities.^9^, ^10^, ^27^ Volunteer service providers' concerns centred around expertise, burden and potential cost. This agrees with the findings of Shandra^10^ that volunteer organisations hesitate to recruit people with chronic illness because of the appropriate equipment, training or transportation costs entailed. Considering this, there was agreement that OTs could be the interface between pwNC and volunteering service providers to protect volunteer services from being overburdened and the pwNC from being overwhelmed. Both OTs and pwNC seemed to find this interface obvious and suitable, and pwNC expressed trust in the OTs to assess appropriate voluntary work.

However, OTs identified potential barriers to implementation, such as agreement of supervisors and pragmatic considerations such as scheduling appointments and if adaptations were required. The OTs also raised the issue of the financial cost of this support. While community participation interventions are cost‐effective,^12^ there is a recognition that infrastructure and funding are required. Indeed, the outpatient nurses expressed concern about the potential misuse of volunteering in the health system. Nevertheless, community participation is recommended in the transition process from hospital to home for people with chronic illness,^12^ and it is recommended that agencies fund voluntary services and educate stakeholders about the benefits.^28^ It has previously been found that pwNC were treated unequally during the volunteering process.^10^ This study found factors to support accessibility that included simplified application and cancellation procedures, varying group sizes, sufficient information and the establishment of a buddy system. The OTs believed that if a volunteer's requirements and ability are aligned, extensive (time) resources would not be required.

There was also a concern that there was a risk to pwNC of being overworked. Shandra^10^ advocated the possibility of teamwork to compensate for their changing ability to perform. Theis et al.^29^ explored energy‐saving strategies of pwNC based on prioritising between important and unimportant activities or maintaining the activities that sustain one's livelihood; volunteering in a team could prevent the need for such balancing. This is in line with the findings of Meulenkamp et al.,^30^ who observed that those with chronic illnesses perceive personal control over their health condition as associated with volunteering. Related to this, pwNC were concerned that their performance might be compared to other volunteers, and people may have assumptions about their ability to perform tasks, sense of responsibility and reliability. PwNC emphasised focussing on the person's intention to give their 100% and not only on performance. Indeed, Shandra^10^ found pwNC were more generous in spending time volunteering.

Several limitations should be considered. The outpatient nurses could not contribute as much as the other participants, largely due to the COVID‐19 pandemic, and only four focus groups were conducted. While we had a robust rationale for conducting separate focus groups for pwNC and professionals, this should also be considered. Furthermore, it is important to note that while participants in the focus group agreed on many aspects, the study was not designed to find consensus.

The group of pwNC was heterogeneous in terms of their conditions, and it is important to reaffirm that five of six had the perspective of someone who volunteers. Exploring the impact of different types of neurological conditions on volunteering experiences was beyond the scope of this study. While this study developed ideas and perspectives for future practice, future studies may wish to compare or explore perspectives according to diagnoses. In addition, the majority did not live alone and were car owners. Macdonald et al.^31^ found that volunteers with chronic illness often were single and home and car owners, and importantly, the cost of travel was identified as a barrier to volunteering.

When assessing generalisability, it is important to consider that the study was carried out in Germany and within the context of its health policies and resources.

## 5 CONCLUSION

The results of this study support the therapeutic potential of volunteering for pwNC. The stakeholders who took part fundamentally agreed on the value of supporting pwNC to volunteer. However, they also raised concerns about current structures, burden and cost. A recent scoping review on community participation found only barriers reposted in the literature.^12^ Therefore, the potential facilitators, such as information and systems to match suitability, flexibility and expectations, identified in the current study are important and may help to inform future interventions. Further research is required to evaluate interventions to confirm the beneficial effects and inform implementation.

## CONFLICT OF INTEREST STATEMENT

The authors declare no conflict of interest.

## Supporting information

Supporting information.

## Data Availability

The data sets used and analysed during the current study are available from the corresponding author upon reasonable request.

## References

[1] Marková A . The “inclusive volunteering” phenomenon: research into the volunteering of people with disabilities. Contact. 2018;20(1):48‐56. 10.1016/j.kontakt.2017.10.003

[2] Hill W , Macartney M . The role of occupational therapy in enabling people with chronic pain to return to work or education. Anaesth Intensive Care Med. 2019;20(8):443‐445. 10.1016/j.mpaic.2019.05.007

[3] Keen C , Hashmi‐Greenwood M , York J , Armstrong IJ , Sage K , Kiely D . Exploring a physiotherapy well‐being review to deliver community‐based rehabilitation in patients with pulmonary hypertension. Pulm Circ. 2019;9(4):1‐9. 10.1177/2045894019885356 PMC6831978 31723408

[4] Blickem C , Kennedy A , Vassilev I , et al. Linking people with long‐term health conditions to healthy community activities: development of Patient‐Led Assessment for Network Support (PLANS). Health Expect. 2013;16(3):48‐59. 10.1111/hex.12088 PMC3908360 23731452

[5] Klinedinst NJ , Resnick B . The Volunteering‐In‐place (VIP) Program: providing meaningful volunteer activity to residents in assisted living with mild cognitive impairment. Geriatr Nurs. 2016;37(3):221‐227. 10.1016/j.gerinurse.2016.02.012 26975836

[6] Moffatt S , Steer M , Lawson S , Penn L , O'Brien N . Link worker social prescribing to improve health and well‐being for people with long‐term conditions: qualitative study of service user perceptions. BMJ Open. 2017;7(7):e015203. 10.1136/bmjopen-2016-015203 PMC5541496 28713072

[7] World Health Organization . Towards a common language for functioning, disability and health. ICF. 2023. Retrieved June 28, 2023, from https://cdn.who.int/media/docs/default-source/classification/icf/icfbeginnersguide.pdf?sfvrsn=eead63d3_4&download=true

[8] Bandura A . Self‐Efficacy: The Exercise of Control. W.H. Freeman and Company; 1997.

[9] Marella M , Devine A , Armecin GF , Zayas J , Marco MJ , Vaughan C . Rapid assessment of disability in the Philippines: understanding prevalence, well‐being, and access to the community for people with disabilities to inform the W‐DARE project. Popul Health Metr. 2016;14:26. 10.1186/s12963-016-0096-y 27489509 PMC4971707

[10] Shandra CL . Disability and social participation: the case of formal and informal volunteering. Soc Sci Res. 2017;68:195‐213. 10.1016/j.ssresearch.2017.02.006 29108597

[11] Skivington K , Matthews L , Simpson SA , et al. A new framework for developing and evaluating complex interventions: update of Medical Research Council guidance. BMJ. 2021;374:n2061. 10.1136/bmj.n2061 34593508 PMC8482308

[12] Gough C , Baker N , Weber H , et al. Integrating community participation in the transition of older adults from hospital to home: a scoping review. Disabil Rehabil. 2022;44(17):4896‐4908. 10.1080/09638288.2021.1912197 33909534

[13] Rentenbescheid24.de . Die haeufigsten Ursachen der Erwerbsminderung [The most common causes of reduction in earning capacity]. Halle (Saale), Jena, Rain (G). 2021. Retrieved January 9, 2021, from https://rentenbescheid24.de/die-haeufigsten-ursachen-der-erwerbsminderung/

[14] Tong A , Sainsbury P , Craig J . Consolidated Criteria for Reporting Qualitative Research (COREQ): a 32‐item checklist for interviews and focus groups. Int J Qual Health Care. 2007;19(6):349‐357. 10.1093/intqhc/mzm042 17872937

[15] Morgan DL , Scannell AU . Deciding on group size. Planning Focus Groups. SAGE Publications Inc; 1998. 10.4135/9781483328171.n7

[16] Saunders B , Sim J , Kingstone T , et al. Saturation in qualitative research: exploring its conceptualization and operationalization. Qual Quant. 2018;52:1893‐1907. 10.1007/s11135-017-0574-8 29937585 PMC5993836

[17] Krueger RA . Developing Questions for Focus Groups. Vol 1‐3. SAGE Publications Inc.; 1998. 10.4135/9781483328126

[18] Stewart DW , Shamdasani PN , Rook DW . Analysing focus group data. Focus Groups. SAGE Publications; 2007.

[19] Braun V , Clarke V . Using thematic analysis in psychology. Qual Res Psychol. 2006;3(2):77‐101. 10.1191/1478088706qp063oa

[20] Lamnek S . Qualitative Sozialforschung. Beltz Verlag; 2010.

[21] Attride‐Stirling J . Thematic networks: an analytic tool for qualitative research. Qual Res. 2001;1(3):385‐405. 10.1177/146879410100100307

[22] Okun MA , August KJ , Rook KS , Newsom JT . Does volunteering moderate the relation between functional limitations and mortality? Soc Sci Med. 2010;71(9):1662‐1668. 10.1016/j.socscimed.2010.07.034 20864238 PMC2975672

[23] Leigh‐Hunt N , Bagguley D , Bash K , et al. An overview of systematic reviews on the public health consequences of social isolation and loneliness. Public Health. 2017;152:157‐171. 10.1016/j.puhe.2017.07.035 28915435

[24] Kielhofner G . Model of Human Occupation: Theory and Application. Lippincott Williams & Wilkins; 2008.

[25] Wilcock AA . Reflections on doing, being and becoming. Aust Occup Therap J. 1999;46:1‐11. 10.1046/j.1440-1630.1999.00174.x

[26] Vaportzis E , Niechcial MA , Gow AJ . A systematic literature review and meta‐analysis of real‐world interventions for cognitive ageing in healthy older adults. Ageing Res Rev. 2019;50:110‐130. 10.1016/j.arr.2019.01.006 30707947

[27] Hammel J , Magasi S , Heinemann A , Whiteneck G , Bogner J , Rodriguez E . What does participation mean? An insider perspective from people with disabilities. Disabil Rehabil. 2008;30(19):1445‐1460. 10.1080/09638280701625534 18923977

[28] Rak EC , Spencer L . Community participation of persons with disabilities: volunteering, donations and involvement in groups and organisations. Disabil Rehabil. 2016;38(17):1705‐1715. 10.3109/09638288.2015.1107643 26681543

[29] Theis KA , Murphy L , Hootman JM , Helmick CG , Sacks JJ . Arthritis restricts volunteer participation: prevalence and correlates of volunteer status among adults with arthritis. Arthritis Care Res. 2010;62(7):907‐916. 10.1002/acr.20141 20597117

[30] Meulenkamp T , Rijken M , Cardol M , Francke AL , Rademakers J . People with activity limitations' perceptions of their health condition and their relationships with social participation and experienced autonomy. BMC Public Health. 2019;19(1):1536. 10.1186/s12889-019-7698-9 31744483 PMC6862750

[31] Macdonald W , Kontopantelis E , Bower P , Kennedy A , Rogers A , Reeves D . What makes a successful volunteer Expert Patients Programme tutor? Factors predicting satisfaction, productivity and intention to continue tutoring of a new public health workforce in the United Kingdom. Patient Educ Couns. 2009;75(1):128‐134. 10.1016/j.pec.2008.09.024 19041212

