# Supplementary material for: Exploring the barriers and facilitators to volunteering as an intervention for those with long‐term neurological conditions: How make therapeutic volunteering possible?
Source: Health Expect. 2023 Oct 15;27(1):e13891. doi: 10.1111/hex.13891 (PMC10726151; doi:10.1111/hex.13891)
Supplement: Supplementary file 1 — Supporting information. [file HEX-27-e13891-s001.docx]

**Supplement 1**

| **Quotes listed** | **English** | **German** |
| --- | --- | --- |
| Basic Theme 1:  Self-Efficacy | *“Volunteering means you can do what you can do.” (Nr. C2)* | “Ehrenamt bedeutet ja man kann das machen was man auch kann.” |
|  | *“I lost cycling with the onset of my illness. Cycling had always been crucial for me […], I went to the fair in [town] and discovered the special bikes, the recumbent bikes […] I was so enthusiastic about it and then founded the private initiative [name].” (Nr. C1)* | “Ich habe das Fahrradfahren verloren mit dem Auftreten meiner Krankheit. Das Fahrradfahren war fuer mich immer sehr wichtig gewesen, …. und bin dann zur Messe nach [Stadt] gekommen und habe die Spezial-Raeder, die Liege-Raeder entdeckt […] Ich bin so begeistert davon gewesen und habe dann die Privatinitiative [Name] gegruendet.” |
|  | *“Of course, sometimes it’s fun when you see the results […] A lot has to be done in advance before you really meet together. I find that amazing.” (Nr. P5)* | “Also klar, manchmal macht das auch Spass, wenn man das Ergebnis sieht […] Da muss im Vorfeld einiges gemacht werden, bevor man sich wirklich gemeinsam treffen kann. Das finde ich schon erstaunlich.” |
| Basic Theme 2:  New Social Role | *“I think we would be doing a great service to society if someone came to do voluntary work and made a contribution  […] it would perhaps change the social image of people with disabilities a little bit.” (Nr. C3)* | “Ich denke, wir wuerden schon einen grossen gesellschaftlichen Dienst leisten, wenn jemand kommt um im Ehrenamt taetig zu sein und da seinen Beitrag leistet […] es wuerde das gesellschaftliche Bild von Menschen mit Behinderung vielleicht ein kleines Stueck veraendern.” |
| Basic Theme 3: Vulnerability | *“Yes, I think that the state rests too much on the fact that you could use volunteers for certain tasks because it's just cheaper.” (Nr. P5)* | “Ja, ich denke, dass der Staat sich zu sehr darauf ausruht, dass man ja fuer gewisse Aufgaben Ehrenamtler nehmen koennte, weil es ist halt guenstiger.” |
|  | *“My speed at the moment I do something [sic]should be allowed to be my speed, and that would be good for society as a whole if we all slowed down a bit.” (Nr.C1)* | “Sprich meine Geschwindingkeit ist in dem Moment wo ich tue, soll dann auch meine Geschwindigkeit sein duerfen und das wuerde der ganzen Gesellschaft gut tun, wenn wir alle so ein bisschen zurueck gehen, bisschen in einer vertraeglichen Geschwindigkeit machen.” |
| Basic Theme 4: Flexibility and Simplification | *“They might say I can do half an hour a day or an hour a week, or I want three hours a day because I need the structure. […] That these people [pwNC] don't feel this pressure from the beginning, like in the real working world.” (Nr. P2)* | “Die koennten vielleicht sagen, ich schaffe eine halbe Stunde am Tag oder eine Stunde in der Woche oder sie sagen ich moechte aber jeden Tag 3 Stunden, weil ich die Struktur brauche. […] Dass diese Leute nicht von vornherein diesen Druck empfinden, wie in der realen Arbeitswelt.” |
|  | *“Especially in spring, there is the possibility to grow plants on the windowsill, to do it for yourself, but to know what you are doing it for [sic].” (Nr. P5)* | “Da gibt es gerade im Fruehjahr die Moeglichkeit da zieht man Pflanzen auf der Fensterbank an, macht da das fuer sich, aber weiss fuer was man es macht.” |
| Basic Theme 5: Available Information | *“They can also make descriptions of their work under other aspects so far, but there you might have to add fields like "flexible in terms of time" or "up to three hours per week", "barrier-free", or "without a lift" or "you have to be able to do this and that!” (Nr. C3)* | “Bislang machen sie vielleicht Beschreibungen, was sie so tun, aber da muesste man vielleicht noch Felder ergaenzen wie “zeitlich flexible” oder “bis zu drei Stunden die Woche”, “barrierefrei” oder “ohne Aufzug” oder “Du musst das und das koennen!”.” |
| Basic Theme 6:  Fixed Contact Person | *“I think it always comes down to having a coordinator you can talk to.” (Nr. C3)* | “Ich denke es haengt immer daran, dass man einen Koordinatoren hat, den man ansprechen kann.” |
| Basic Theme 7:  Social attitude | *“For me as a disabled person, it is more important […], that you think about the fact that you can get into this situation yourself.” (Nr. C4)* | “Fuer mich als Behinderten ist es eigentlich wichtiger, den ersten Punkt zu sehen, dass man sich Gedanken macht, dass man selbst in diese Situation geraten koennte.” |
|  | *“I think appreciation is critical […] we should appreciate each other […] I have a past, I have a lot of know-how, I have a lot of potential from my work so far, which is hidden, which I may not be able to access at the moment.”   (Nr. C1)* | “Wertschaetzung finde ich extremst wichtig […] wir sollten uns ueberhaupt hier in der Gesellschaft und sowieso grundsaetzlich und fuer chronisch Kranke umso mehr eine Wertschaetzung […] Ich habe ja eine Vergangenheit, ich habe ja, ich habe ganz viel Know-How, ich habe ein grosse Potenzial aus meiner Taetigkeit bisher, das verschuettet ist, das ich vielleicht gerade nicht abrufen kann.” |
| Basic Theme 8: Learning Atmosphere | *“Don't challenge the beginning [sic] too much by setting the requirements so high, but start with smaller requirements, but then also "throw the child in at the deep end and say, now do this, and then we'll see if we can build on it".” (Nr. 4)* | “Also die Initiative nicht zu stark herausfordern indem man die Ansprueche so hoch steckt, sondern mit kleinern Anspruechen anfangen, aber dann auch “das Kind ins kalte Wasser schmeissen und sagen, jetzt mach das mal und dann gucken wir weiter, ob man aufbauen kann”.” |
| Basic Theme 9:  Social support | *“The solution always requires a group or a supporter.” (Nr. C6)* | “Immer gehoert zu der Loesung eine Gruppe oder ein Unterstuetzer.” |
| Basic Theme 10: Structural support | *“This pressure of having to deliver a certain performance, of being measured by it, then the pressure is gone.” (Nr. C1)* | “Und wenn dieser Druck weg, dieser Druck weg ist, eine bestimmte Leistung erbringen zu muessen, daran gemessen werden.” |
| Organising Theme 1: Impact of Volunteering | *“Then we come to the disadvantages […] envy and jealousy have already been mentioned, but then also the demand for expertise and energy, partly also financial expenditure, too […] that one is left alone with the tasks so that it comes to overload […], but I'm afraid that it's a problem of voluntary work in general and that it doesn't just apply to the chronically ill.” (Nr. C1)* | “Dann kommen wir mal zu den Nachteilen […] Neid und Missgunst wurden schon mal genannt, dann aber auch der Anspruch an Fachkunde und Energie, zum Teil aber auch finanzieller Aufwand […] dass man bei den Aufgaben alleine gelassen wird […] aber ich befuerchte, dass es generell ein Ehrenamtsproblem ist und es nicht nur auf chronisch Kranke zutrifft.” |
|  | *“But there are also unreliabilities [sic] in healthy people. I know some people  […] who have more absences than working hours.” (Nr. C5)* | “Aber es gibt auch Unzuverlaessigkeiten bei gesunden Personen. Also ich kenne einige […] die haben mehr Fehlzeiten als Arbeitszeiten.” |
| Organising Theme 2: Individualisation | *“And that it can reach a level relatively quickly, depending on what you want to offer, where you say: "Oops, that has nothing to do with "part-time" anymore. This is a real job that you do on the side. Not everyone can afford that.” (Nr. P5)* | “Und das es relativ schnell, je nach dem was man anbieten moechte, einen Umfang erreichen kann, wo man sagt: “Ups, das hat nichts mehr mit “nebenher” zu tun. Es ist eigentlich ein richtiger Job, den man noch so nebenher erledigt. Das kann sich nicht jeder leisten.” |
| Organising Theme 3: Developmental Space | *“You always have to find out that you don't set the tasks too high or too low. So that they [the tasks] can still offer satisfaction and success.” (Nr. C5)* | “Man muss dann immer rausfinden, dass man die Aufgaben nicht zu hoch und nicht zu niedrig ansetzt. Also dass sie [die Aufgabe] noch Befriedigung und Erfolg bieten koennen.” |
| Organising Theme 4: Funded supported volunteering | *“But it would also be nice if there was someone from the community […] because the service provider himself can't always afford to guide the people.” (Nr. P2)* | “Aber es waere auch schoen, wenn es von der Stadt jemanden gaebe […] weil der Dienstleister selbst es nicht immer leisten kann die Leute einzuweisen.” |
| Global Theme | *“People might find it better to do something than to be placed somewhere where they play board games or things like that. That has been food for thought for me.” (Nr. P2)* | “Die Leute faenden es vielleicht besser, sie wuerden eine Taetigkeit verrichten, als dass sie irgendwo untergebracht waeren, wo sie Gesellschaftsspiele machen oder solche Sachen. Das ist fuer mich ein Denkanstoss gewesen.” |
|  | *“Is a person concerned at all willing to allow transparency, or does he prefer to keep his difficulties behind him. I see the potential for difficulties there.” (Nr. P1)* | “Ist ein Betroffener ueberhaupt bereit dazu Transparenz zuzulassen oder will er lieber mit seinen Schwierigkeiten hinter dem Berg halten. Da sehe ich Schwierigkeitspotenzial.” |
|  | *“I think that it would help everyone. If you say: "Flexibility is not possible for me; I need reliable people", then I know as someone who refers or recommends the job: "Okay, it's not suitable for this [person]". (Nr. P3)* | “Ich denke damit waere allen geholfen. Wenn Sie sagen: “Bei mir ist mit Flexibilitaet nichts moeglich, ich brauche zuverlaessige Leute”, dann weiss ich als jemand, der den Job weitervermittelt oder empfiehlt: “Okay hierfuer ist er [Person] nicht geeignet”.” |
|  | *“I think that taking care of the chronically ill person would probably be reduced to an adjustment of the workplace; rather, it would not even be necessary in case of doubt if we were to choose the right one [workplace].” (Nr. P3)* | “Ich denke das sich Kuemmern um den chronisch Kranken wuerde sich wahrscheinlich in dem Fall auf eine Anpassung des Arbeitsplatzes reduzieren; vielmehr waere im Zweifel gar nicht noetig, wenn wir das Passende (Ehrenamt) aussuchen wuerden.” |
